# Draft Genome Sequence of Raoultella ornithinolytica Strain HH3

**DOI:** 10.1128/genomeA.00270-18

**Published:** 2018-04-12

**Authors:** Hongsheng Huang, Marc-Olivier Duceppe, Beverley Phipps-Todd

**Affiliations:** a Canadian Food Inspection Agency, Ottawa Laboratory-Fallowfield, Ottawa, Ontario, Canada

## Abstract

Raoultella ornithinolytica is a Gram-negative, nonmotile, encapsulated, and aerobic bacillus and an emerging hospital-related bacterial pathogen of humans. Here, we report a 5,977,517-bp draft genome sequence for Raoultella ornithinolytica strain HH3, isolated from a pretreatment sample collected at a Canadian wastewater treatment facility.

## GENOME ANNOUNCEMENT

Raoultella ornithinolytica is a Gram-negative, nonmotile, encapsulated, and aerobic bacillus belonging to the family *Enterobacteriaceae* (1). R. ornithinolytica usually inhabits aquatic environments and is an emerging pathogen causing hospital-acquired infections in humans, mostly systemic infections and urinary tract infections (2). This article reports the draft genome sequence of R. ornithinolytica strain HH3, isolated from a pretreatment sample collected at a Canadian wastewater treatment facility using the MFHPB-19 method (3) for the isolation of Escherichia coli. Species determination was conducted using commercial biochemical test kits (Remel Micro ID, Oxoid, and API-20E, bioMérieux, Inc., Canada) and confirmed using whole-genome sequencing.

The isolate was kept in brain heart infusion broth containing 25% glycerol at −80°C following the isolation procedure. For sequencing, genomic DNA was extracted from overnight cultures grown at 37°C on tryptic soy agar using the Qiagen DNeasy blood and tissue DNA purification kit (Qiagen, Inc., Toronto, Ontario, Canada) and quantified using Qubit 3.0 (Life Technologies, Inc., Burlington, Ontario, Canada). The sequencing library was constructed using the Nextera XT DNA library preparation kit (Illumina, Inc., San Diego, CA, USA), and the library quality was analyzed using BioAnalyzer (Agilent Technologies, Santa Clara, CA, USA). The library was sequenced with the MiSeq platform (Illumina, Inc.) using the version 3 sequencing kit. A total of 1,491,678 paired-end reads (300 bp) were generated.

Adapter sequences and low-quality bases were trimmed (bbduk), and overlapping pairs were merged (bbmerge) using the BBtools software suite (http://jgi.doe.gov/data-and-tools/bbtools/). Preprocessed reads were assembled using SPAdes version 3.10.1 (4) and polished with Pilon version 1.22 (5). The assembly resulted in 79 contigs (>200 bp), with an average of 65-fold genome coverage. Contigs were ordered with Mauve version 2015-02-13 (http://darlinglab.org/mauve/mauve.html) using R. ornithinolytica strain A14 (NCBI reference sequence no. NZ_CP008886), the genome closest to R. ornithinolytica strain HH3 according to Mash version 1.1.1 (6). The length of the draft genome was 5,977,517 bp, with a GC content of 55.5%. Sixteen of the 79 contigs show homology with plasmid sequences of related species. Gene predictions and annotations were performed using NCBI Prokaryotic Genome Annotation Pipeline (7), which predicted 5,941 genes, 5,825 coding sequences (CDSs), 79 tRNAs, and 1 clustered regularly interspaced short palindromic repeat (CRISPR) array. Thirteen prophage sequences were detected by PHASTER (8).

### Accession number(s).

This whole-genome shotgun project has been deposited in DDBJ/ENA/GenBank under the accession no. NKYI00000000. The version described in this paper is the first version, NKYI01000000.
